# Carbon-Efficient Scheduling in Fresh Food Supply Chains with a Time-Window-Constrained Deep Reinforcement Learning Model

**DOI:** 10.3390/s24237461

**Published:** 2024-11-22

**Authors:** Yuansu Zou, Qixian Gao, Hao Wu, Nianbo Liu

**Affiliations:** 1 University of Electronic Science and Technology of China, Chengdu 611731, China; yuansuzou@std.uestc.edu.cn (Y.Z.); gaoqixian@std.uestc.edu.cn (Q.G.); wu__hao@std.uestc.edu.cn (H.W.); 2 Xihua University, Chengdu 610039, China

**Keywords:** Intelligent Transportation Systems, fresh food supply chain, reinforcement learning, carbon-efficient scheduling

## Abstract

Intelligent Transportation Systems (ITSs) leverage Internet of Things (IoT) technology to facilitate smart interconnectivity among vehicles, infrastructure, and users, thereby optimizing traffic flow. This paper constructs an optimization model for the fresh food supply chain distribution route of fresh products, considering factors such as carbon emissions, time windows, and cooling costs. By calculating carbon emission costs through carbon taxes, the model aims to minimize distribution costs. With a graph attention network structure adopted to describe node locations, accessible paths, and data with collection windows for path planning, it integrates to solve for the optimal distribution routes, taking into account carbon emissions and cooling costs under varying temperatures. Extensive simulation experiments and comparative analyses demonstrate that the proposed time-window-constrained reinforcement learning model provides effective decision-making information for optimizing fresh product fresh food supply chain transportation and distribution, controlling logistics costs, and reducing carbon emissions.

## 1. Introduction

Climate change is a pressing global challenge, largely due to greenhouse gas emissions from economic activities. In response, numerous nations have introduced regulations to reduce these emissions. These initiatives include setting emission caps, carbon offset programs, carbon trading schemes, and carbon taxes, all aimed at cutting greenhouse gases and their environmental damage. China exemplifies this approach, with widespread adoption of carbon management and trading policies. In 2013, a pilot emission trading system was introduced in seven regions. By 2017, the National Development and Reform Commission officially established a national carbon market, with a plan to expand its reach over time. As policies for carbon emissions become more prevalent and the carbon market expands, businesses, particularly those in energy-intensive industries like the fresh food supply chain, are confronted with the crucial task of addressing carbon emission limitations [1,2].

Fresh food supply chain is a low-temperature supply chain system that integrates the refrigeration industry with logistics. In recent years, with the improvement of living standards, China’s fresh food supply chain industry has experienced significant growth. As of 2023, the market size of China’s fresh food supply chain reached RMB 637.1 billion, and it is expected to expand to RMB 868.6 billion by 2025. The market growth rate for the fresh food supply chain in 2023 was 15.55%, with an anticipated growth of 17.61% by 2025. Additionally, the total demand for a fresh food supply chain in 2023 is approximately 3.5 billion tons, reflecting a growth of 6.1%. This growth highlights the important role of fresh food supply chains in meeting the evolving needs of consumers and the industry, with the Chinese market currently accounting for about 25% of the global cold chain market. However, although its development has promoted economic development to a certain extent, it has also imposed a serious burden on the environment. Compared with ordinary logistics, a fresh food supply chain requires the use of refrigeration equipment (such as refrigerated trucks) to maintain the freshness of products, and normal operation consumes a lot of energy and produces a lot of carbon emission. Therefore, in the context of strict government carbon regulations and consumers’ growing expectations for environmental protection, it has become necessary to incorporate carbon emission reduction into logistics management operational decisions. Since then, how to make operational decisions, reduce carbon emission from cold chain activities, and promote the sustainable development of enterprises has become a thorny issue that needs to be solved urgently.

With the increasing requirements for food freshness and quality, the importance of fresh supply chain management in the logistics field has become increasingly prominent, especially in China, which has huge market demand and complex supply chain networks [3,4]. However, fresh supply chain management faces a series of challenges, especially in cold storage site selection, delivery route optimization, and meeting customer time window restrictions and carbon emission [5]. First, enterprises need to consider cost-effectiveness in the selection of limited cold storage addresses. The geographical location and operating costs of cold storage are key factors when choosing [6,7,8]. Secondly, the optimization of distribution routes must not only meet cost-effectiveness, but also consider carbon footprint [9]. Enterprises must carry out refined management in distribution planning and route optimization to reduce carbon emissions during transportation, including choosing appropriate transportation modes, optimizing vehicle scheduling and loading efficiency. Furthermore, the complexity brought by the diversified storage of fresh products also needs to consider carbon emissions [10,11,12]. Meat and vegetables have different requirements for transportation conditions. Transport vehicles under different temperature settings not only affect costs but also energy consumption and carbon emission [13,14]. Enterprises need to evaluate the transportation costs and environmental impacts under different temperature settings and seek a balance between cost and environmental protection. China’s fresh food transportation law stipulates the storage and transportation temperature requirements for different fresh products. According to the “14th Five-Year Plan for fresh food supply chain Development” and “Fruit and Vegetable Cold Chain Circulation Specifications”, meat products should be kept at a specified temperature environment during transportation. Although the specific temperature is not clearly specified, the refrigerated transportation temperature of meat is usually 0 °C to 4 °C, and the frozen transportation temperature is below −18 °C [15]. There are different classifications of cold chain circulation temperature ranges for fresh fruits and vegetables [16]. For example, the temperature range of Class A fruits and vegetables is 0 °C to 4 °C, while the temperature of quick-frozen fruits and vegetables should be below −18 °C [17,18]. This is directly related to the design, operating costs and energy consumption level of transport vehicles.

With China’s commitment to carbon peak and carbon neutrality goals, the fresh food supply chain industry is facing the pressure of emission reduction and the opportunity of transformation. Enterprises need to explore low-carbon technologies and operating models, such as the use of new energy vehicles, optimization of logistics network design, and improvement of logistics efficiency, to achieve green development and long-term sustainable development. This study aims to explore how fresh food supply companies in China can formulate reasonable cold storage site selection strategies and optimize distribution routes under limited resource conditions, while meeting the temperature requirements of different fresh products, customer time window restrictions, and considering the impact of carbon emission, so as to achieve comprehensive optimization of cost-effectiveness, customer satisfaction and environmental sustainability [19,20].

## 2. Literature Review

The core purpose of this paper is to use reinforcement learning technology to obtain the optimal path planning in the cold chain transportation process. This study comprehensively considers the carbon emission generated in each link of the entire transportation process, while taking into account the time window constraints and the formulation of route plans, and fully considers the impact of economic factors and carbon emission regulations [21,22]. Therefore, this study mainly reviews the relevant literature in three areas: one is the application of reinforcement learning in the field of path planning; the second is the fresh food supply chain planning model that integrates environmental protection concepts and considers carbon emission issues; the third is to consider the customer time window and satisfaction, as well as the path planning model that considers the actual application scenarios.

### 2.1. RL-Related Path Planning Model

In our study, we first focused on using reinforcement learning to build a logistics planning model for fresh food supply chains. Reinforcement learning was chosen because it has shown excellent ability to handle complex decision-making problems. As an adaptive learning algorithm, reinforcement learning can learn through interaction with the environment to achieve long-term optimal decisions [23,24]. This feature is particularly critical in the field of logistics planning because logistics networks usually contain a large number of nodes, face complex demand changes, and cope with a changing transportation environment [25]. By training a unified policy model, reinforcement learning can capture reward signals and follow feasibility rules to provide near-optimal solutions for a wide range of problem instances. In addition, unlike algorithms that need to be retrained for each new problem instance, reinforcement learning models can generate a series of continuous action decisions in real time, thereby solving problems quickly and reliably without retraining for each new instance. This ability makes reinforcement learning a powerful tool for optimizing fresh food supply chain distribution routes, effectively controlling logistics costs, and significantly reducing carbon emissions [26].

Compared with the above studies, our reinforcement learning model introduces an attention graph to better express the logistics delivery location and time window constraints. The integrated reinforcement learning model shows higher flexibility and adaptability when dealing with complex distribution environments in solving path planning problems.

### 2.2. Carbon-Efficient Scheduling Model

Since route optimization of fresh food transportation is conducive to the low-carbon cold chain logistics, many studies evaluate and minimize the environmental impact of the delivery process by implementing carbon emission constraints. A standard and objective methodology of wine supply chain [10] is proposed for calculating carbon footprint with life cycle assessment methodology. Carbon emission control is considered in cold chain logistics of fresh agricultural products [11], in which Geographically Weighted Regression and Multi-scale Geographically Weighted Regression methods are used to determine the impact of regional carbon emissions. In human-driven edge computing, a basic decision-making model for the supply chain under the carbon tax constraint [12] compares and analyzes the optimal decision-making problem of the supply chain between the centralized and decentralized decisions of producers and retailers under the carbon tax constraint. A distribution cost model [13] is constructed to solve the cold chain logistics problem, according with the effect of temperature changes on the decay rate of fresh products during unloading, the carbon emission costs during transportation and cold storage, customer satisfaction, as well as the traffic situation of the actual distribution route.

Compared with the above logistics planning research, our research considers the impact of temperature restrictions under real regulations on route planning, which can more accurately evaluate and reduce the environmental impact of the transportation process. Our model is designed to be flexible and user-friendly, and can adapt to logistics needs of all sizes, from local distribution to wider regional networks  [27]. Moreover, we combine the path planning problem with the warehouse location problem, effectively solving the common resource allocation and cost control problems in logistics planning scenarios, and providing support for achieving sustainable logistics operations for fresh food supply industry.

### 2.3. Practical Application Value Model

In this study, we delve into the specific challenges of cold chain logistics distribution, particularly the temperature sensitivity of fresh produce and the time window constraints of customer service [28]. By considering these key factors, our approach aims to increase distribution efficiency, personalization and satisfaction of customer service. Compared with the existing literature, the advantage of this study is that it is oriented to practical applications and provides a practical solution for the implementation of the cold chain logistics industry, so as to directly promote the positive change of the industry, rather than a simple theoretical discussion [29]. In addition, this study focuses specifically on the field of cold chain logistics, where, by optimizing distribution routes and warehouse locations, our approach not only contributes to reducing carbon emissions and responding to the global quest for environmentally sustainable green development strategies but also provides a practical way for businesses to go green [30]. The methodology combines cost-effectiveness, customer satisfaction and environmental sustainability to provide a comprehensive and innovative perspective for this study in the field of cold chain logistics, ensuring its significant value at both theoretical and practical levels.

## 3. Model Building

This study addresses a realistic fresh food supply chain issue, providing path planning for fresh food supply companies to ensure the lowest cost calculated with carbon emission. The company provides transportation services to some customers within a city, and each customer has strict time window requirements for fresh food deliveries, with financial penalties for late arrivals. We plan the routes to serve all customers once within a day as agreed, to calculate transportation costs. Furthermore, considering the significant impact of the starting point (fresh food warehouse) location on transportation costs, the company can optimize the location selection. That is, for a limited number of available warehouses within a city, transportation path planning is made based on all available starting points to select the optimal warehouse that minimizes transportation costs.

**Figure 1:**
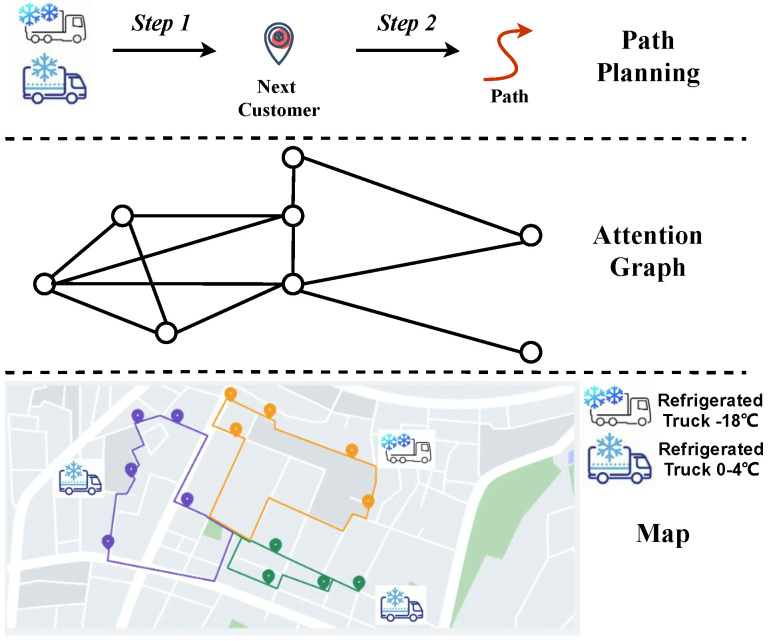
Overview of fresh food supply environment.

As shown in Figure 1, some customer nodes are deployed in a city area, whose positions are fixed and can be precisely determined. These customer nodes are visited by the refrigerated trunks with different temperatures for fresh food supply. We utilize an abstract attention graph to map out the system’s nodes and potential routes. Each customer node has defined data and collection windows that act as constraints for visit order. Because of urban mapping constraints, the graph only includes existing paths, limiting route planning to these routes. We have created a two-step route planning method for refrigerated trucks to streamline planning within a smaller state space. First, we pick the next customer node from those available to be the immediate destination. Second, we guide the trucks to take a viable route to get to that destination.

Different from traditional path optimization problems, this study not only considers the distance cost factor, but also the impact of carbon tax policies on carbon emission costs. By using reinforcement learning to optimize path planning, our model can dynamically learn and adapt to changing transportation conditions and realize real-time optimization of transportation routes to reduce carbon emission and refrigeration costs. At the same time, the model conducts temperature sensitivity analysis to provide customized transportation solutions for fresh products with different storage requirements to ensure their quality and safety, while optimizing energy use and reducing environmental impact. In addition, the model also considers the impact of ambient temperature on transportation costs and carbon emission, has ambient temperature adaptability, and can adjust transportation strategies under different climatic conditions to achieve optimal transportation efficiency and minimum environmental impact. Finally, the model provides decision makers with a comprehensive evaluation perspective through a comprehensive cost–benefit analysis, helping them make sustainable and environmentally friendly transportation decisions while ensuring transportation efficiency [31].

### 3.1. Model Constraints

- Find a set of truck routes $S=\{s_{1},\ldots ,s_{K}\}$ that satisfy the following conditions:
  1. Each customer node is visited exactly once, and each route starts and ends at the warehouse node.
  2. Each route $s_{k}$ satisfies the truck’s load capacity constraint, i.e., $\sum_{i\in s_{k}}q_{i}\leq Q$.
  3. Each route $s_{k}$ must complete the service within the time window $\left[a_{i},b_{i}\right]$ of the associated customer i.

### 3.2. Explanation of Symbols

Define the graph G={V,E,q,c,a,b}, where

- *V* is the set of vertices, containing N+M+1 nodes, where *M* is the number of alternative warehouse nodes, 0 is the currently selected warehouse node, and nodes 1,…,N are customer nodes.
- *E* is the set of edges, containing all node pairs {i,j} in *V*, where i,j∈V and i≠j.

Each node *i* has the following attributes:

- Euclidean coordinates $r_{i}\in R^{2}$.
- Demand $q_{i}>0$, with $q_{0}=0$ for the warehouse node.

The weights $c_{ij}$ on edge *E* represent the transportation cost from node *i* to node *j*, here expressed in time units, including

- The time cost of actual travel distance.
- Carbon emission cost, which can be considered as an additional cost proportional to travel distance or time.
- Refrigeration cost, the energy cost associated with maintaining the required temperature for goods, which may be related to travel time and the efficiency of the truck’s refrigeration system.

Each customer node *i* has a time window $\left[a_{i},b_{i}\right]$, where $a_{i}\leq b_{i}$, representing the time range during which customer *i* can accept service.

The time window $\left[a_{0},b_{0}\right]$ for warehouse node 0 represents the earliest and latest times a truck can depart and return to the warehouse.

The service time required for each customer *i* is $h_{i}$.

There are *K* homogeneous trucks, each with the same load capacity Q>0.

The notations are shown as Table 1.

### 3.3. Distribution Cost Analysis

1. Fixed costs

The fixed costs consist of two main components: the fixed cost of delivery trucks and the cost of warehouse rental. The total fixed cost can be expressed as

(1) $$C_{\mathrm{fix}}=\sum_{k\in K}F_{k}+\sum_{m\in M}R_{m}$$

2. Shipping costs

The transportation cost is directly proportional to the distance traveled by the trucks. The total transportation cost is given by

(2) $$C_{\mathrm{tran}}=\sum_{k\in K}\sum_{\{i,j\}\in E}f\cdot d_{ij}\cdot x_{ijk}$$

The unit distance transportation cost f can be expressed as

(3) f=ρ·Cr

3. Cooling costs

Cooling cost consists of three main components: the refrigeration cost during transportation, the refrigeration cost during the waiting period, and the refrigeration cost during the loading and unloading process. The total cooling cost can be expressed as

(4) $$C_{\mathrm{cool}}=C_{\mathrm{RC},\mathrm{trans}}+C_{\mathrm{RC},\mathrm{wait}}+C_{\mathrm{RC},\mathrm{load}}$$

- The refrigeration cost during transportation is denoted by $C_{\mathrm{RC},\mathrm{trans}}$:
  (5) $$C_{\mathrm{RC},\mathrm{trans}}=\sum_{k\in K}\sum_{\{i,j\}\in E}v_{k}\cdot d_{ij}\cdot \eta \left(T_{k}\right)\cdot x_{ijk}$$
- The refrigeration cost during the waiting period is denoted by $C_{\mathrm{RC},\mathrm{wait}}$:
  (6) $$C_{\mathrm{RC},\mathrm{wait}}=\sum_{k\in K}\sum_{i\in V}w_{i}\cdot e_{k}\cdot \eta \left(T_{k}\right)\cdot y_{ki}$$
- The refrigeration cost during the loading and unloading process is denoted by $C_{\mathrm{RC},\mathrm{load}}$:
  (7) $$C_{\mathrm{RC},\mathrm{load}}=\sum_{k\in K}\sum_{i\in V}h_{i}\cdot m_{k}\cdot \eta \left(T_{k}\right)\cdot z_{ki}$$

The temperature efficiency factor $\eta (T_{k})$ is defined based on the temperature requirements of the refrigerated truck, reflecting the difference in energy consumption needed to maintain the required temperature for goods at different temperatures.

For a refrigerated truck at 4 °C, $\eta (T_{k})$ can be set to 1. For a refrigerated truck at −18 °C, $\eta (T_{k})$ can be set to 1.5.

4. Carbon emission costs

Carbon emission costs are related to fuel consumption during truck driving and energy consumption during refrigeration. Carbon emission costs are composed of the costs associated with carbon emission during driving, the carbon cost of cooling while driving, and the carbon emission costs during service. The total carbon emission cost can be represented as the sum of the following individual components [32,33,34,35,36]:

(8) $$C_{\mathrm{total}-\mathrm{emission}}=C_{\mathrm{drive}-\mathrm{emission}}+C_{\mathrm{RC}-\mathrm{emission}}+C_{\mathrm{RC}-\mathrm{service}}$$

- Carbon emission costs during driving:
  (9) $$C_{\mathrm{drive}-\mathrm{emission}}=\sum_{k\in K}\sum_{\{i,j\}\in E}cp\cdot e_{\mathrm{drive}}\cdot d_{ij}\cdot x_{ijk}$$

The carbon emission consumption $e_{drive}$ per unit distance can be calculated using the following formula:

(10) $$e_{\mathrm{drive}}=\rho \cdot \phi$$

b. Carbon costs of cooling while driving:
  (11) $$C_{\mathrm{RC}-\mathrm{emission}}=\sum_{k\in K}\sum_{\{i,j\}\in E}cp\cdot e_{\mathrm{refrig}}\cdot t_{ij}\cdot x_{ijk}$$
c. Carbon emission costs during service:
  (12) $$C_{\mathrm{RC}-\mathrm{service}}=\sum_{k\in K}\sum_{i\in V}cp\cdot e_{\mathrm{service}}\cdot h_{i}\cdot y_{ki}$$

5. Penalty Cost

In the field of fresh food supply chains, when the goods cannot be delivered within the time requested by the customer, a certain penalty cost must be paid. The time window penalty cost for each customer *i*, denoted by $C_{\mathrm{time}-\mathrm{penalty},i}$, can be represented by a piecewise function:

(13) $$C_{\mathrm{time}-\mathrm{penalty},i}=\left\{\begin{array}{cc} \alpha \cdot (a_{i}-T_{\mathrm{arrival},i}) & \mathrm{if}\;T_{\mathrm{arrival},i}<a_{i}\\ 0 & \mathrm{if}\;a_{i}\leq T_{\mathrm{arrival},i}\leq b_{i}\\ \;\mathrm{Large}\;\mathrm{Penalty} & \mathrm{if}\;T_{\mathrm{arrival},i}>b_{i} \end{array}\right.$$

Here, $a_{i}$ and $b_{i}$ represent the start and end times of the time window for customer *i*, respectively, and $T_{\mathrm{arrival},i}$ is the actual time when the truck arrives at customer *i*. Parameter α is the penalty cost per unit time for early arrival. In fresh food supply chains, timely delivery is crucial. Therefore, in order to ensure extremely high timeliness of delivery, we have set strict punishment measures for late arrivals beyond the customer’s scheduled time window. See Table 2.

The total time window penalty cost, denoted by $C_{\mathrm{total}-\mathrm{time}-\mathrm{penalty}}$, is calculated as

(14) $$C_{\mathrm{total}-\mathrm{time}-\mathrm{penalty}}=\sum_{i\in V}C_{\mathrm{time}-\mathrm{penalty},i}$$

### 3.4. Model Implement

**Figure 2:**
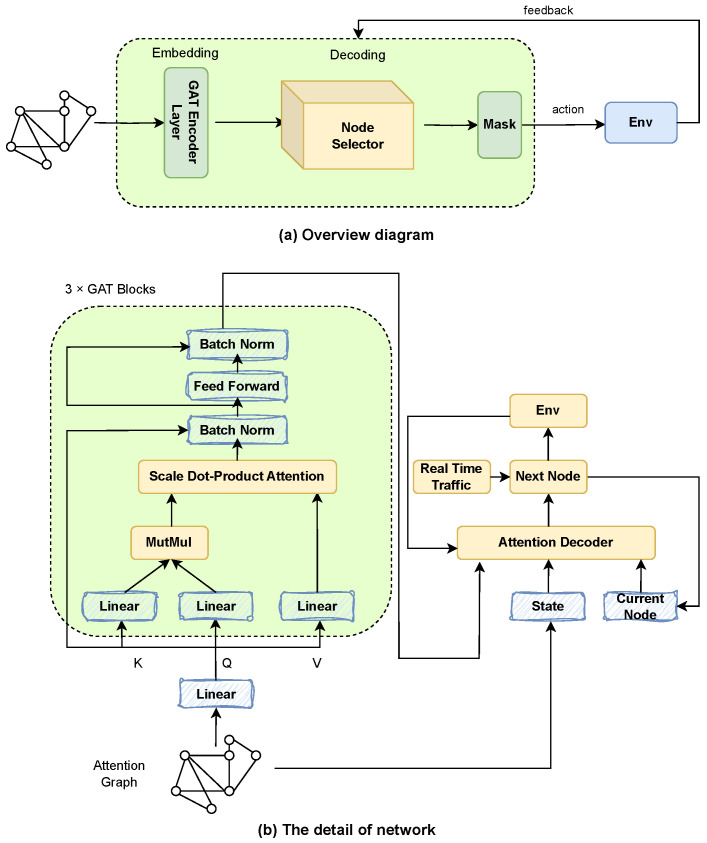
The proposed RL framework.

Figure 2a shows an overview of the proposed model, which includes a graph attention encoder and a node selection decoder. The graph attention encoder is responsible for embedding the input attention graph information into the feature vector, and the node selector uses the embedded information to select the next node to be accessed. When selecting nodes, it is necessary to meet the constraints of the corresponding time window and load capacity. After executing the action, the environment will provide feedback on the result, and the node selector will continue to select the next node based on the current state until the end.

As shown in Figure 2b, the graph attention encoder first obtains the input two-dimensional coordinates, node demand, and node time window. It maps the node dimension to the embedding dimension. The encoder consists of three layers of graph attention layers, which extract high-order features of nodes layer by layer to describe the complex relationships between nodes in the model. Generate the current state based on the input of the problem, which includes information such as the current node, visited nodes, and used capacity. In each step of solution construction, calculate the logarithmic probability and mask of the next set of nodes for the current node. If the decoding strategy is greedy, directly select the node with the highest probability. If the decoding strategy is sampling, select the next visited node based on probability, and then update the state to a new state, adding the visited nodes to the sequence. In addition, the lower-level graph neural network uses a fixed abstract expression of distance and time window, and the actual second routing considers real-time traffic changes, which are dynamic and adaptive.

Before describing the algorithm, we first define the model.

The state space is divided into two parts: vehicle state and node state. The vehicle state includes the current remaining capacity, route length, current position, visited node set, vehicle speed, etc. The node state includes the location coordinates of the node, the demand for the node, and the time constraint of the node. The dynamic and static elements in the state are considered separately.

The action space is defined as the next node that the vehicle chooses to visit at the current time step. When selecting an action, the model will make decisions based on the vehicle and node states, combined with the time window and capacity constraints.

The reward function comprehensively considers factors such as transportation cost, refrigeration cost, and carbon emission cost, and it is defined as the negative value of the sum of their costs, aiming to minimize the cost of cold chain transportation. In order to reflect the global performance of the path planning problem, the reward function uses the cumulative cost after the sequence is completed as a negative reward instead of a single-step reward. This design can avoid local optimal solutions and improve the optimization effect of global performance.

(15) $$R_{i}=\left\{\begin{array}{cc} -(C_{\mathrm{tran},i}+C_{\mathrm{cool},i}+C_{\mathrm{total}-\mathrm{emission},i}+C_{\mathrm{time}-\mathrm{penalty},i}) & \mathrm{On}\;\mathrm{time}\;\mathrm{or}\;\mathrm{early}\;\mathrm{arrival}\\ -1000 & \mathrm{Lately}\;\mathrm{arrival} \end{array}\right.$$

We use the Monte Carlo policy gradient method to train the model and define the cumulative reward obtained from the initial state as a performance metric to optimize the overall performance of the strategy. To reduce the variance of gradient estimation, we introduce a baseline method based on Rollout. Specifically, we first randomly initialize the policy π and use it to infer the randomly generated training set instances to obtain the cumulative return value corresponding to each instance. Then, we estimate the gradient of the loss function relative to the trainable parameters based on these returns and use the Adam optimizer to update the model parameters. When training the model, we use sample decoding to enhance the model’s exploration ability and avoid falling into local optimality, while in Rollout we use greedy decoding to provide a more stable reference value. The loss function is defined as follows [37]:

(16) $$\nabla L_{RL}\left(\theta |x\right)=E_{P_{\theta }\left(\pi |x\right)}\left(G_{t}^{sample}-G_{t}^{greedy}\right)\nabla \log P_{\theta }\left(\pi |x\right)$$

We have implemented our algorithm, as shown in Algorithm 1.
**Algorithm 1** Reinforce with baseline (Rollout).**Require:** 
Initial network weight θ, Input Instance *x***Ensure:** 
<IsDone,∑cost,OutputSequence P>    1:**repeat**    2:   Generate corresponding action $a_{t}$ through attention networks that consider traffic conditions    3:   **if** Decode strategy = greedy **then**    4:       Choose the point with the highest probability    5:       Rollout provides stable reference values based on determined strategies    6:   **else if** Decode strategy = sample **then**    7:       Select nodes based on probability value sampling    8:       The model enhances exploration capabilities through sampling    9:   **end if**  10:   Execute action $a_{t}$ and observe new observation $s_{t}$  11:   Put the node selected by action $a_{t}$ at into the Sequence *P*  12:   τ←τ+1  13:**until** Service success or failure  14:Obtain the cumulative return $G_{t}^{greedy}$ of the model under the Rollout baseline  15:Obtain the cumulative return $G_{t}^{sample}$ of the model under the sampling strategy  16:$\theta_{t+1}\leftarrow \theta_{t}+\alpha \left(G_{t}^{sample}-G_{t}^{greedy}\right)\nabla_{\theta }\log P_{\theta }\left(\pi |x\right)$  17:**return**$\;<IsDone,G_{t}^{greedy},P>$

## 4. Experiment Results

To verify the effectiveness of the model, we will test 10000 randomly generated instances and compare the computational performance of various heuristic strategies and our reinforcement learning scheme under different client node numbers, evaluating the performance of the two methods in dealing with problems of different scales. Through experiments, we found that reinforcement learning models can find better solutions in a short period of time when there are a large number of nodes. Then, we sampled 70 instances of nodes and compared the differences in route selection and cost between reinforcement learning and guided local search. Finally, we considered the actual logistics scenario based on the model mentioned above, randomly selected the locations of four warehouse nodes, and used reinforcement learning methods to calculate the transportation cost within one day to select the optimal warehouse location.

### 4.1. Experimental Settings

#### 4.1.1. Map and Data

When considering the impact of traffic fluctuations on fresh food transportation, our map model adopts a log normal distribution to describe the driving speed of vehicles between any two nodes. This distribution is appropriate because it can generate positive velocity values and simulate changes in traffic speed in the real world, which is crucial for ensuring the freshness and timely delivery of food [38].

(17) $$f\left(v\left(t\right)\right)=\frac{1}{\sqrt{2\pi v\left(t\right)\sigma_{v}}}e^{-\frac{{\left(\ln v\left(t\right)-\mu \right)}^{2}}{2\sigma_{v}^{2}}}$$

The average value μ of the distribution represents the expected driving speed under stable traffic conditions, which is predicted based on historical data and traffic models. This value reflects the average speed that vehicles can expect to achieve without unexpected traffic delays. The standard deviation $\sigma_{v}$ measures the variability of driving speed, revealing the uncertainty of driving speed caused by traffic fluctuations. During periods of traffic congestion, the actual driving speed may be significantly lower than the average μ; during smooth traffic, the speed may approach or slightly exceed this value.

This definition can enable our map model to better capture and respond to the impact of traffic fluctuations on fresh food transportation, thereby improving logistics efficiency and food quality.

We generated instances containing 20, 30, 50, 70, and 100 nodes (clients), where the distance between cities was calculated using the two-dimensional Euclidean distance formula. The goal is to minimize the total distance traveled by vehicles, where the coordinates of customer nodes and warehouse nodes are evenly distributed within the range of [0, 1]. Each customer’s demand is randomly sampled within the integer range 1.. 9, and the vehicle capacity is fixed at 30, 35, 40, 45, and 50 based on the number of nodes. The time window is set between [0, 8], with the left time window randomly selected from [0, 2, 4, 6], while the right time window is fixed as the left time window plus 2. These settings aim to create a diverse and challenging dataset for evaluating the performance of different optimization algorithms.

#### 4.1.2. Training Settings

The model was implemented using PyTorch 1.12.1, and we ran the experiments on an Ubuntu 20.04.4 LTS server with an NVIDIA GeForce RTX 3080 graphics card. The hyperparameters used for model training are shown in Table 3.

In the training process of the reinforcement learning model, we first generate a training dataset under the same distribution and initialize the policy network based on the attention mechanism, with the goal of maximizing the cumulative return obtained from a fixed initial state $S_{0}$. In order to reduce the variance of the gradient estimate and accelerate the convergence of the model, we introduced a Rollout-based method as a baseline. By generating a Monte Carlo estimate under the current state and policy, we obtain a more stable reference value, thereby improving the efficiency and accuracy of training. Compared with the model without the baseline, the model with the baseline performs better.

In terms of hyperparameter selection, we chose a discount rate of 1. This choice is based on the actual needs in the cold chain transportation scenario: since the reference value of short-term returns is low, and optimizing long-term cumulative costs is our main goal, setting the discount rate to 1 can better focus on global optimization. In addition, we use a larger batch size to improve the stability of policy updates and use a sampling decoding strategy during training to enhance the exploration ability of the model, helping it to search the policy space more effectively and avoid falling into local optimal solutions.

### 4.2. Comparison with Baselines

We compared our deep reinforcement learning scheme to some classic search strategies to evaluate the performance of fresh food supply chains, with OrTools as a famous Google optimization tool. In OrTools, we compared various local search strategies and generated 10,000 instances from the same data distribution. We used deep reinforcement learning (DRL), guided local search (GLS) [39], simulated annealing (SA) [40], and TABU search [41] to find the optimal solution to the problem.

The following Table 4 shows the comparison between the deep reinforcement learning algorithm and three heuristic algorithms when the number of customer nodes is 20, 30, 50, 70, and 100, respectively. The entries in the table represent the proportion of instances where deep reinforcement learning methods outperform the corresponding heuristic algorithms among 10,000 instances. It can be seen that when the network size is small, heuristic solutions perform similarly to deep reinforcement learning methods, and heuristic solutions can obtain solutions in a shorter time. However, deep reinforcement learning models outperform these heuristic algorithms when there are more nodes. As the network size increases, the computational speed of heuristic algorithms decreases rapidly, while the deep reinforcement learning algorithms still exhibit good performance.

### 4.3. Illustrative Movement Traces

**Figure 3:**
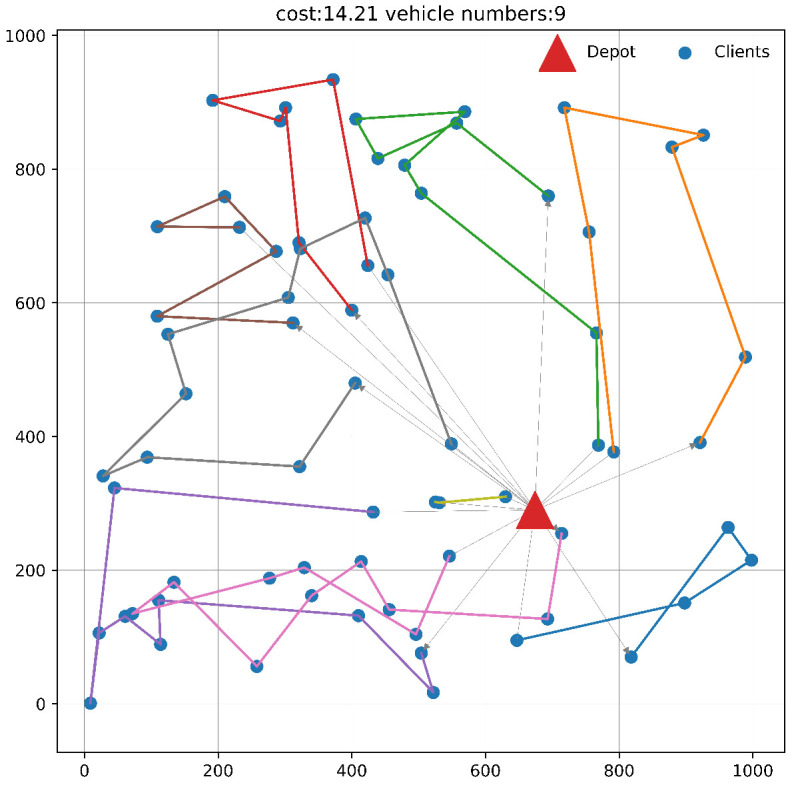
Path planning using the DRL algorithm.

**Figure 4:**
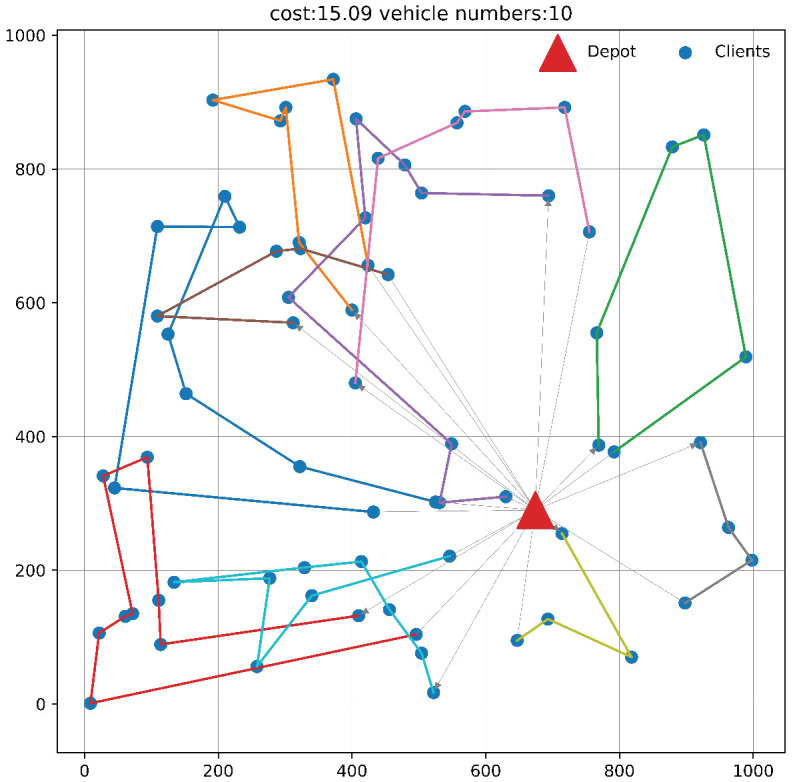
Path planning using the GLS algorithm.

We used the reinforcement learning algorithm and the OrTools local guided search algorithm to solve a sampled instance with 70 nodes. The vehicle’s movement trajectory is shown in Figure 3 and Figure 4, respectively, and detailed information on the trajectory is listed in Table 5 below. The coordinates, time window, and node capacity information of the customer instance node are also listed in Table 6. The node index range is 1 to 70, the coordinates of the warehouse node are [0.674, 0.291], and the warehouse is located at index 0.

Each refrigerated truck needs to start from the warehouse and return to the warehouse after completing the visit to the corresponding node. In order to complete the visit to each node, the reinforcement learning algorithm needs to deploy 9 refrigerated trucks, with a total distance cost of 14.21; the local guided search algorithm requires 10 refrigerated trucks, with a total distance cost of 15.09. It can be seen that when the network scale is large, as the number of nodes increases, the possible path integration and vehicle scheduling solutions grow exponentially, the heuristic algorithm is prone to fall into the local optimal solution, and the reinforcement learning algorithm is more efficient in vehicle scheduling and cost control.

### 4.4. Warehouse Location Selection

In this section, we use DRL to select the optimal location for the warehouse. We first selected an instance with 70 nodes, where the customer nodes’ locations are the same as in the original instance, and there are four available warehouse nodes, which are randomly generated using a uniform distribution. The cost of each refrigerated truck is determined based on the truck’s capacity, and the total delivery time is 8 h. The customer nodes’ time windows are selected from [0, 2], [2, 4], [4, 6], and [6, 8]. According to the model’s definition, transportation costs, carbon emission costs, and refrigeration costs are all taken into account. The objective function values are calculated for each selected warehouse, and the optimal warehouse location is compared. The customer nodes’ coordinates, time windows, and demand quantities are shown in Table 6, and the parameters related to refrigerated transportation are listed in Table 2. The average driving speed of the truck is set at 32 km per hour, fuel consumption is 0.377 L per kilometer, the daily usage cost of the truck is 100, the daily rental cost of the warehouse is CNY 1000, the fuel price is CNY 7.25 per liter, and the carbon tax price is CNY 1 per kilogram.

By randomly selecting four coordinates as starting points, we calculated the logistics costs and route planning results for the warehouse for one day. According to the data analysis in Table 7, the best warehouse location is [0.504, 0.818], while the worst warehouse location is [0.480, 0.920]. The optimized route planning can save CNY 78.9 in one day, accumulating to CNY 28,798.5 per year, which corresponds to a reduction of 28.8 tons of carbon emissions. This indicates that optimizing the location of fresh food warehouses not only has significant cost–benefits but also aligns with emission reduction targets.

## 5. Conclusions

This study presents an innovative optimization model for cold chain logistics distribution routes, integrating time window constraints and reinforcement learning to achieve carbon-efficient scheduling within fresh food supply chains. By simultaneously considering carbon emissions, time windows, and cooling costs, our model optimizes distribution routes to reduce logistics costs and minimize environmental impacts. Extensive simulation experiments and comparative analyses validate the effectiveness of our model, demonstrating that it outperforms traditional heuristic algorithms, particularly for large-scale node problems. Furthermore, this research explores how optimizing warehouse locations can reduce logistics costs and carbon emissions in real-world logistics scenarios, further demonstrating the practical application potential of our model.

Future research should focus on integrating real-time data for dynamic route optimization, exploring multi-objective frameworks to balance efficiency and sustainability, and assessing the long-term sustainability of the model under varying conditions. Additionally, investigating the impact of policy changes on route planning and applying these methods to other logistics domains can further contribute to the field.

## Figures and Tables

**Table 1:** Relevant parameters of the models.

Notations	Description
*F*	represents the fixed cost for each truck.
*K*	denotes the total number of available trucks.
*k*	represents a truck that is in use.
$R_{m}$	represents the rent for each warehouse.
*m*	represents the currently rented warehouse.
*M*	denotes the set of available warehouses.
*f*	represents the transportation cost per unit distance.
$d_{ij}$	is the distance from node *i* to node *j*.
$v_{k}$	The fuel consumption rate of truck *k* at $T_{\mathrm{env}}$.
$x_{ijk}$	Indicates if truck *k* transports between customers *i* and *j*.
$w_{i}$	The waiting time at customer *i*.
$e_{k}$	The energy consumption rate of truck *k* at $T_{\mathrm{env}}$.
$\eta (T_{k})$	The adjustment factor for truck *k*’s energy rate at $T_{k}$.
$y_{ki}$	Indicates if truck *k* waits at customer *i*.
$h_{i}$	The service time at customer *i*.
$z_{ki}$	Indicates if truck *k* loads/unloads at customer *i*.
$e_{\mathrm{drive}}$	Carbon emission cost per unit distance.
$e_{\mathrm{refrig}}$	Carbon emission rate for truck *k* during driving.
$t_{ij}$	The actual driving time from node *i* to node *j*.
$e_{\mathrm{service}}$	Carbon emission cost per unit time for the refrigeration system.
α	The penalty cost for early arrival per unit time.
$a_{i}$ and $b_{i}$	Time window boundaries for customer *i*.
$T_{\mathrm{arrival},i}$	The arrival time of the truck at customer *i*.
ρ	The fuel consumption per unit distance when the vehicle is running.
Cr	The price of one liter of fuel.
ϕ	The coefficient value of CO_2_ emission.
cp	The unit carbon trading price.

**Table 2:** Relevant parameters of the models.

Parameters	Values
ρ	0.377 L/km
F	100 CNY
R	1000 CNY
Cr	7.25 CNY/L
ϕ	2.63 kg/L
cp	1 CNY/kg
$v_{k}$	20 CNY/h
$m_{k}$	15 CNY/h
$e_{\mathrm{drive}}$	0.992 kg/km
$e_{\mathrm{refrig}}$	3.1625 kg/h
$e_{\mathrm{service}}$	4.5 kg/h
α	300 CNY/h

**Table 3:** Hyperparameter values for the attention model.

Parameters	Attention Model
Batch size	512
Episode	100
Learning rate	0.0001
Embedding dimension	128
Hidden dimension	128
N encoder layers	3
Discount Rate	1

**Table 4:** Average shortest distance of four methods on various instances.

Number of Nodes	DRL	GLS	SA	TABU
20	6.983	6.923	6.997	6.998
30	8.904	9.022	9.067	9.046
50	12.448	12.814	12.804	12.803
70	16.187	17.399	17.049	17.176
100	19.440	29.784	29.565	29.566

**Table 5:** Comparison of GLS and DRL on examples.

Guided Local Search
truck 1: 0→48→38→47→67→57→51→3→61→49→0
truck 2: 0→37→64→36→45→54→10→2→0
truck 3: 0→27→66→41→16→59→33→0
truck 4: 0→6→70→46→62→68→29→39→44→15→69→0
truck 5: 0→24→23→22→25→31→8→56→1→63→0
truck 6: 0→18→5→58→7→50→0
truck 7: 0→35→52→60→28→40→4→0
truck 8: 0→13→30→55→42→0
truck 9: 0→53→34→17→65→0
truck 10: 0→11→9→26→20→14→32→19→12→43→21→0
Cost: 15.09
Deep Reinforcement Learning
truck 1: 0→34→30→55→42→65→0
truck 2: 0→13→59→41→16→40→4→33→0
truck 3: 0→24→60→52→25→28→22→23→66→27→0
truck 4: 0→37→64→36→45→54→10→2→0
truck 5: 0→9→11→6→46→70→39→44→15→61→49→0
truck 6: 0→18→5→58→57→3→51→0
truck 7: 0→53→17→26→20→43→12→32→29→19→14→69→21→0
truck 8: 0→35→38→62→68→47→67→8→7→31→50→56→0
truck 9: 0→48→1→63→0
Cost: 14.21

**Table 6:** Instance data from Sample 7.

Sample instance for 7:
Customer corrdinates: [[0.532, 0.302], [0.424, 0.656], [0.11, 0.714], [0.756, 0.707], [0.109, 0.581],
+ [0.411, 0.133], [0.323, 0.682], [0.306, 0.609], [0.504, 0.077], [0.373, 0.934], [0.522, 0.018],
+[0.258, 0.056], [0.921, 0.392], [0.329, 0.204], [0.01, 0.002], [0.926, 0.852], [0.694, 0.127], [0.313, 0.571],
+ [0.278, 0.188], [0.415, 0.213], [0.547, 0.221], [0.479, 0.806], [0.504, 0.765], [0.695, 0.761], [0.406, 0.875],
+ [0.456, 0.141], [0.769, 0.387], [0.569, 0.886], [0.073, 0.136], [0.964, 0.264], [0.42, 0.728], [0.135, 0.182],
+ [0.793, 0.377], [0.819, 0.071], [0.405, 0.48], [0.301, 0.893], [0.4, 0.589], [0.322, 0.355], [0.061, 0.132],
+ [0.719, 0.893], [0.88, 0.834], [0.899, 0.151], [0.341, 0.163], [0.022, 0.107], [0.294, 0.873], [0.112, 0.156],
+ [0.152, 0.464], [0.525, 0.303], [0.432, 0.288], [0.454, 0.643], [0.233, 0.713], [0.439, 0.817], [0.714, 0.255],
+ [0.193, 0.903], [0.998, 0.216], [0.549, 0.389], [0.211, 0.76], [0.287, 0.677], [0.989, 0.52], [0.558, 0.869],
+ [0.046, 0.324], [0.095, 0.369], [0.63, 0.31], [0.322, 0.691], [0.647, 0.095], [0.767, 0.555], [0.126, 0.554],
+ [0.028, 0.342], [0.496, 0.104], [0.115, 0.089]]
Time window:[[4.0, 6.0], [6.0, 8.0], [6.0, 8.0], [6.0, 8.0], [0.0, 2.0], [0.0, 2.0], [4.0, 6.0], [4.0, 6.0],
+ [0.0, 2.0], [6.0, 8.0], [0.0, 2.0], [4.0, 6.0], [0.0, 2.0], [4.0, 6.0], [6.0, 8.0], [4.0, 6.0], [2.0, 4.0],
+ [0.0, 2.0], [4.0, 6.0], [2.0, 4.0], [6.0, 8.0], [4.0, 6.0], [4.0, 6.0], [2.0, 4.0], [4.0, 6.0], [2.0, 4.0],
+ [4.0, 6.0], [4.0, 6.0], [4.0, 6.0], [2.0, 4.0], [4.0, 6.0], [4.0, 6.0], [6.0, 8.0], [0.0, 2.0], [2.0, 4.0],
+ [0.0, 2.0], [0.0, 2.0], [2.0, 4.0], [2.0, 4.0], [6.0, 8.0], [4.0, 6.0], [4.0, 6.0], [4.0, 6.0], [4.0, 6.0],
+ [2.0, 4.0], [0.0, 2.0], [4.0, 6.0], [2.0, 4.0], [6.0, 8.0], [4.0, 6.0], [6.0, 8.0], [2.0, 4.0], [0.0, 2.0],
+ [6.0, 8.0], [2.0, 4.0], [4.0, 6.0], [4.0, 6.0], [2.0, 4.0], [4.0, 6.0], [2.0, 4.0], [6.0, 8.0], [2.0, 4.0],
+ [6.0, 8.0], [0.0, 2.0], [4.0, 6.0], [4.0, 6.0], [4.0, 6.0], [2.0, 4.0], [6.0, 8.0], [0.0, 2.0]]
Data size: [9, 9, 5, 9, 7, 2, 8, 8, 3, 5, 9, 3, 6, 5, 7, 5, 2, 9, 2, 4, 2, 2, 5, 6, 3, 2, 1, 6, 4, 1,
+ 3, 2, 8, 4, 9, 6, 4, 1, 4, 1, 5, 4, 7, 1, 3, 5, 3, 6, 8, 1, 8, 9, 3, 8, 5, 1, 7, 8, 6, 2, 1, 4, 8, 8, 5, 2, 2, 5, 8, 4]

**Table 7:** Solution found for Sample 7.

Position1 [0.504,0.818]	
Fixed costs: 1800	Shipping costs: 480.43
Cooling costs: 232.39	Carbon emission costs: 1283.85
Total costs: 3796.67	
Position2 [0.480,0.920]	
Fixed costs: 1800	Shipping costs: 520.47
Cooling costs: 239.26	Carbon emission costs: 1315.84
Total costs: 3875.57	
Position3 [0.682,0.690]	
Fixed costs: 1800	Shipping costs: 495.21
Cooling costs: 234.93	Carbon emission costs: 1295.65
Total costs: 3825.79	
Position4 [0.693,0.380]	
Fixed costs: 1800	Shipping costs: 490.27
Cooling costs: 234.08	Carbon emission costs: 1291.71
Total costs: 3816.06	

## Data Availability

Data will be made available upon request.
